# Multi-Layer Identification of Highly-Potent ABCA1 Up-Regulators Targeting LXRβ Using Multiple QSAR Modeling, Structural Similarity Analysis, and Molecular Docking

**DOI:** 10.3390/molecules21121639

**Published:** 2016-11-29

**Authors:** Meimei Chen, Fafu Yang, Jie Kang, Xuemei Yang, Xinmei Lai, Yuxing Gao

**Affiliations:** 1 College of Chemistry and Chemical Engineering, Fujian Normal University, Fuzhou 350007, Fujian, China; 2 College of Traditional Chinese Medicine, Fujian University of Traditional Chinese Medicine, Fuzhou 350122, Fujian, China; meinuohua2007@163.com (J.K.); mei_tcm@163.com (X.Y.); keerer1990@163.com (X.L.); 3 College of Chemistry and Chemical Engineering, Xiamen University, Xiamen 361005, Fujian, China; gaoxingchem@xmu.edu.cn

**Keywords:** ABCA1, QSAR, molecular modeling, similarity analysis, molecular docking, LXRβ

## Abstract

In this study, in silico approaches, including multiple QSAR modeling, structural similarity analysis, and molecular docking, were applied to develop QSAR classification models as a fast screening tool for identifying highly-potent ABCA1 up-regulators targeting LXRβ based on a series of new flavonoids. Initially, four modeling approaches, including linear discriminant analysis, support vector machine, radial basis function neural network, and classification and regression trees, were applied to construct different QSAR classification models. The statistics results indicated that these four kinds of QSAR models were powerful tools for screening highly potent ABCA1 up-regulators. Then, a consensus QSAR model was developed by combining the predictions from these four models. To discover new ABCA1 up-regulators at maximum accuracy, the compounds in the ZINC database that fulfilled the requirement of structural similarity of 0.7 compared to known potent ABCA1 up-regulator were subjected to the consensus QSAR model, which led to the discovery of 50 compounds. Finally, they were docked into the LXRβ binding site to understand their role in up-regulating ABCA1 expression. The excellent binding modes and docking scores of 10 hit compounds suggested they were highly-potent ABCA1 up-regulators targeting LXRβ. Overall, this study provided an effective strategy to discover highly potent ABCA1 up-regulators.

## 1. Introduction

Atherosclerosis is a disease that results from the accumulation of lipid-rich plaques within the walls of large arteries, and has become one of the most common causes of death worldwide. Epidemiological studies firmly confirmed an inverse relationship between serum high-density lipoprotein-cholesterol (HDL-C) levels and the incidence of atherosclerosis. The main antiatherogenic mechanism of HDL is transporting excess cholesterol out of macrophages by reverse cholesterol transport [1]. The ATP-binding cassette transporters A1 (ABCA1) played a critical role in reverse cholesterol transport, which mediates the rate-controlling step in HDL particle formation, and substantially affects whole body cholesterol and HDL metabolism and the development of atherosclerosis [2,3]. Dysfunction of ABCA1 or mutations in ABCA1 gene leads to increased atherogenesis [4]. Inhibition of ABCA1 degradation was found to promote HDL biosynthesis and exhibited antiatherogenesis [5]. Thus, the discovery of up-regulators of ABCA1 expression may provide a promising strategy of antiatherogenic therapy.

Recently, a novel series of flavonoids-based ABCA1 up-regulators have been reported by Bu et al. [6]. However, their up-regulation role in ABCA1 expression mostly remained unclear. It is well-known that ABCA1 expression was transcriptionally regulated by liver X receptors (LXRs) [7]. LXRβ directly binds to the C-terminal region of ABCA1 (2247LTSFL2251) to modulate its post-translational regulation. Thus, a search for highly potent ABCA1 up-regulators targeting LXRβ prompted us to investigate the structure–activity relationship of these ABCA1 up-regulators. Here, multi-layer in silico approaches, including quantitative structure activity relationship (QSAR) methods, structural similarity analysis and molecular docking simulation were performed to guide structural optimization and expound the action mechanism for ABCA1 up-regulators. QSAR models relate the chemical structure to a specific activity or property using many linear and nonlinear algorithms. This method has become increasingly popularity in many fields for predicting compound properties, e.g., toxicity prediction, physical property prediction, and biological activity prediction [8]. Structural similarity analysis plays a significant role in many aspects of chemoinformatics, including similarity searching, virtual screening, synthesis design, and property prediction [9]. Molecular docking simulation has been successful in prioritizing large chemical libraries to identify experimentally-active compounds and widely used to understand binding modes and important interactions [10].

To the best of our knowledge, no in silico study has yet been reported to identify highly potent ABCA1 up-regulators so far. In this paper, we attempted to establish highly predictive QSAR classification models as a fast filter for screening highly-potent ABCA1 up-regulators in drug development and also investigate the action mechanism of up-regulating ABCA1 expression. Firstly, different linear and nonlinear classification approaches, including linear discriminant analysis (LDA), support vector machine (SVM), radial basis function neural network (RBFNN), and classification and regression trees (CART), were applied to construct highly-predictive QSAR classification models and mined the structural features responsible for their up-regulation activity of ABCA1. Then, to avoid the limitation or overemphasis of any modeling approach, a consensus QSAR model was initially developed for screening highly potent ABCA1 up-regulator from the ZINC database. To ensure maximum reliability and accuracy of our QSAR models, molecular similarity analysis was also performed to filter compounds in ZINC database compared to known highly-potent ABCA1 up-regulators with the best activity (compound **36**). Only the compounds that fulfilled the requirement of structural similarity of 0.7 were subjected to our QSAR models. Finally, new screened compounds were docking into the LXRβ binding site to understand their role in up-regulating ABCA1 expression and further find molecules targeting LXRβ.

## 2. Results and Discussion

### 2.1. Results of LDA Model

After a stepwise method combined with LDA (SW-LDA) performed, four molecular descriptors were selected from the above remaining 221 descriptors. The corresponding LDA model was simultaneously derived by these descriptors. Table 1 listed the selected descriptors, their chemical meanings, F-test values, Wilks’ lamba values, and standardized coefficients. F-test values, calculated from Wilks’ lambda, are the measure of a descriptor’s importance. Thus, the importance of four descriptors was varied in the order lip_don > vsurf_W2 > a_nCl > vsurf_DD23. The correlation matrix of the selected descriptors was shown in Table 2. All linear correlation coefficient values for each pair of descriptors were less than 0.65, indicating that they were independent [11]. The prediction classification results of LDA model were listed in Table 3. As described in Table 4, the established LDA model was of very successful statistical significance and good predictive ability. The accuracy value of this model revealed that it can give a training accuracy of 90% in predicted classification. The accuracy_LOO_ value was 83.33% (bigger than 50%), indicating that the developed model had good stability and predictive ability. Additionally, the predictive accuracy on the test set was 90.91%, showing the good prediction and generalization ability. Furthermore, sensitivity (SE) and specificity (SP) were calculated to reveal the predictive ability of different classifications of models. In the LDA model, both sensitivity and specificity were 90%, respectively, implying that the LDA model had the same ability to predict the active compounds as the inactive compounds.

### 2.2. Interpretation of Descriptors

The molecular descriptors in the LDA model were allowed to provide some vital structural features to govern ABCA1 up-regulating activity of flavonoids. The model encompassed four descriptors: a_nCl, lip_don, vsurf_DD23, and vsurf_W2. a_nCl represents the number of chlorine atoms. The positive coefficient of this descriptor illustrated the presence of chlorine atoms contributing to greater activity. lip_don is the number of OH and NH atoms. Its negative coefficient indicated that high lip_don tended to attenuate the activity of molecules. Two vsurf_ descriptors, vsurf_W2 and vsurf_DD23, are volume and surface properties, which depend on the structural connectivity and the conformation (their dimensions are measured in Å) of the molecules. These two descriptors explain the interaction of molecules with hydrophobic and hydrophilic parts of the protein through some surface properties such as electrostatic and hydrophobicity [12]. The vsurf_W2 descriptor explains the hydrophilic region of the molecules and is calculated at −0.5 kcal/mol energy levels, which may be defined as the molecular envelope accessible by solvent water molecules. The positive contribution of this descriptor explained that the polarizable property on the van der Waals (vdW) surface of the molecules was important for the interactions. The vsurf_DD23 descriptor signifies the contact distances of vsurf_DDmin, representing the distances, for the OH2 and DRY probes, between the best three local minima of interaction energy when the probes interact with a target molecule [13]. The positive sign of this descriptor illustrated that by increasing the distance between the second-lowest and third-lowest hydrophobic interaction energies would enhance the interaction with the target.

From the above discussion, we can conclude that the presence of chlorine atoms, polarizable groups and contact distances of lowest hydrophobic energy in the molecules were favorable to the bioactivity, while a large number of OH and NH atoms in molecules had a negative effect.

### 2.3. Results of SVM Model

To obtain the best SVM model, the combination of C and γ was optimized by a grid search and every SVM model was validated by leave-one-out (LOO) cross-validation. Generally, the parameter combination with the best LOO cross-validation performance was selected as the optimal set [14]. In this process, the grid search for C and γ over a wide parameter range of 0.001–10,000 tuned in a grid of 100 × 100 was carried out. After 10,000 cycles, the optimum values of C and γ used in the model were obtained (C = 331 and γ = 0.045) with a maximum LOO cross-validation accuracy of 86.67%. Thereby, the final optimal SVM model was generated as well. The corresponding prediction results were listed in Table 3. As shown in Table 4, the SVM model gave the quite satisfactory results: accuracy_train_ of 96.67%, accuracy_LOO_ of 86.67%, accuracy_test_ of 90.91%, sensitivity of 90%, and specificity of 100%, respectively, exhibiting the significantly high prediction and generalization ability.

### 2.4. Results of RBFNN Model

In this study, the input layer consisted of four input neurons and the output layer consisted of one output neuron modeling the up-regulation activity of ABCA1 expression. The number of neurons in the hidden layer required to be optimized to obtain better results. Here, the selection of the optimal hidden center was determined by experiments with a number of trials by taking into account the LOO cross-validation accuracy. The one which gives a maximum LOO cross-validation accuracy is chosen as the optimal value. The maximum LOO cross-validation accuracy was 83.33% and the corresponding number of centers (hidden layer nodes) of RBFN was 18. Then, a 4-18-1 RBFNN model was finally constructed. The corresponding prediction results of the final optimal RBFN model were listed in Table 3. Table 4 contained the resulting values of specificity, sensitivity and separation accuracy of highly potent ABCA1 up-regulators of RBFNN model. It was observed that the RBFNN model was also very successfully established with good prediction ability like the SVM model performed (accuracy_train_ of 96.67%, accuracy_LOO_ of 83.33%, accuracy_test_ of 90.91%, sensitivity of 90% and specificity of 100%, respectively), implying both of them were doing a statistically similar job of yield classification.

### 2.5. Results of CART Model

Similarly, the QSAR model by CART was built using the same input variables as used in the above models. Here, maximum tree depth was set to 5, and the Gini index was used as a basis criteria for splitting nodes into two new groups. The importance sequence of these variables given by the CART model was in the same order as the SW-LDA method did. The predictive classification and statistical results of the CART model were listed in Table 3 and Table 4, respectively. As seen from Table 4, all the prediction accuracy, sensitivity and specificity for the training set reached 100%, respectively. While, the classification accuracy of the test set was 81.82%. So, there was no significant difference in the total classification accuracy among the models of CART, SVM, and RBFNN for the training and test sets. These results indicated that these three models did a comparable job.

### 2.6. Comparison of Different Approaches and Consensus Modeling

From the above discussion, it can be concluded that the developed models performed quite well, and the SVM, RBFNN and CART models slightly outperformed the LDA model based on the same descriptors. As shown in Table 4, the predictive classification performance for the training set by LDA, SVM, ANN, and CART were 90%, 96.67%, 96.67%, and 100%, respectively. The LDA and CART models showed a very good balance between sensitivity and specificity. While the SVM and RBFNN model had a slightly better prediction ability, especially for the low active compounds (higher specificity). As for the test set, the CART model gave the accuracy of 81.82%, while the other three methods can reach 90.91%. It should be noted that although the CART model can give 100% accurate predictions for the training set, the performance of external validation did not show better results than the other models. Thus, it seemed reasonable that a consensus predicted result given by these four kinds of QSAR models might be more correct than individual models, and might, overall, take into account the more peculiar aspects of some particular structure. Here, a consensus QSAR model was derived by averaging the predictions for the dataset given by the four individual models [15]. All of the contributions of the four individual QSAR models were equal and, therefore, we could avoid the limitation or overemphasis of any modeling approach. The statistical results of the derived consensus model were also shown in Table 4. The consensus model produced the train, test, and LOO accuracy of 96.67%, 90.91%, and 83.33%, respectively, and showed a very good sensitivity and specificity of 90% and 100%, respectively.

### 2.7. Screening New Highly-Potent ABCA1 Up-Regulators Targeting LXRβ

After calculating structural similarity between compounds in the ZINC database and compound **36**, compounds with structural similarity below 0.7 were removed from the ZINC database, which resulted in the retrieval of 302 compounds. To ensure the maximum accuracy of predictive classification, the obtained consensus QSAR model was applied to the initial screening potential of ABCA1 up-regulators from these 302 compounds. The compounds with consensus scores of 1 were identified as potential highly-potent ABCA1 up-regulators. Thus, 50 compounds were discovered from these molecules. Then, to better understand their up-regulation mechanism in the expression of ABCA1 and further screen highly-potent ABCA1 up-regulators targeting LXRβ, these 50 compounds were docked into the LXRβ active site using molecular docking in Molecular Operating Environment software (MOE2008.10, Chemical Computing Group Inc., Montreal, QC, Canada).

**Figure 1:**
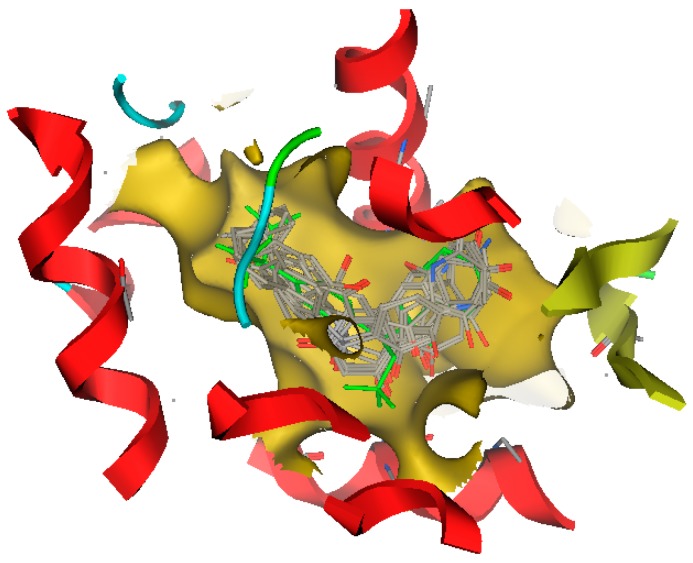
The best-docked conformations and poses of 10 new screened ABCA1 up-regulators in the ligand binding domain of LXRβ: the original ligand of X-ray crystal structure was depicted in green stick mode, newly-screened ABCA1 up-regulators were depicted in gray stick mode.

**Figure 2:**
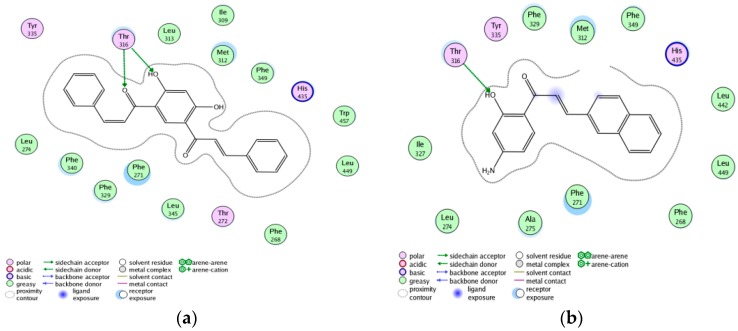
Comparison between the binding mode of ZINC08665430 (**a**) and compound **36** (**b**) in the LXRβ active site.

The ability to reproduce ligands’ X-ray poses in the receptor is a crucial factor to evaluate the effectiveness of docking software [16]. The root-mean-square distance (RMSD) parameter between the ligand in X-ray crystal complex and the redocked ligand is usually calculated to measure the docking accuracy. In our docking study, RMSD was 0.8378 Å, showing that the docking results were very suitable and reliable. Among above 50 discovered compounds, 10 compounds were found to have larger docking scores for LXRβ than the known potent ABCA1 up-regulator (compound **19**), especially five compounds (ZINC08665430, ZINC39949652, ZINC3250227, ZINC05777271, and ZINC32502236) showed higher affinity with LXRβ than compound **36**. The docking results of 10 hit compounds were listed in Table 5. From their structural features and docking scores, we can initially conclude that the electronegative groups in the C6 ring and hydrophobic groups in the C3 chain of chalcones were favorable to the interaction with LXRβ. This can be further explained by their docking modes. The best docked conformations of these 10 compounds were shown in Figure 1, showing that their binding modes were similar to the V pattern of ligand embedded in the X-ray complex of LXRβ. The optimal docked conformation of the most active compound ZINC08665430, as shown in Figure 2a, revealed that ligand recognition was achieved by forming two H-bonds with Tyr316, and producing strong hydrophobic interactions with Met312, Phe349,Trp457, Leu449, Phe268, Leu345, Phe271, Phe329, Phe340, and Leu274 in the active site of LXRβ. Comparative molecular docking between ZINC08665430 and compound **36**, shown in Figure 2, the former had a better docking score than the latter, suggesting that more H-bonds and hydrophobic interactions stabilized the compound within the binding site, thus contributing greater activity. Therefore, these 10 new hit compounds were suggested to be highly potent ABCA1 up-regulators that interacted with LXRβ.

## 3. Materials and Methods

### 3.1. Dataset

In the QSAR analyses, forty one flavonoid-based ABCA1 up-regulators were taken from a dataset from Sun Yat-sen University, China [6]. Their structures and biological activities were listed in Table 3. The compounds of the dataset were categorized as strong or weak activators in terms of their ability to induce the ABCA1 transcription activities, which was normalized to the Renilla luciferase signal (fold change compared to a vehicle control). According to their experimental criteria, the compounds with response ranges of greater than 1.7 were grouped as strong ones (‘1’) and others were grouped as low active ones (‘0’), respectively. To obtain reliable QSAR models, the dataset was split into two subsets by a ratio number of 3:1, a training set of 30 compounds covering a wide variety of structures and a test set of 11 compounds following the distribution of the training set [17]. All 2D structures of compounds in Table 3 were sketched in ChemDraw software and were converted into 3D structures using energy minimization module embedded in MOE (MOE2008.10, Chemical Computing Group Inc.).

### 3.2. Descriptor Calculation and Reduction

MOE offers three class descriptors: 2D descriptors, internal 3D descriptors, and external 3D descriptors, to calculate molecular properties of compounds. Prior to descriptor calculation, the stochastic conformational search was performed to search the optimal geometry conformation of each energy-minimized structure. Only the lowest energy conformer of per structure was subjected to 327 diverse descriptors calculation by utilizing the QSAR module of MOE [18]. To remove redundant information, the descriptors pool was optimized by two criteria. Descriptors with constant or near constant values for all molecules were first deleted from the descriptor pool. Then, only one of the descriptors with high pairwise correlations (correlation coefficient > 0.95) was retained in the descriptor pool [19]. Finally, a total set of 221 descriptors remained and were used for QSAR modeling.

### 3.3. QSAR Modeling Approaches

#### 3.3.1. Stepwise Linear Discriminant Analysis (SW-LDA)

Feature selection is considered as one of the most critical steps for development of QSAR models. In this study, a stepwise method combined with LDA (SW-LDA) was performed to accomplish the selection of the most important descriptors for model construction [20]. LDA looks for a linear discriminant function to divide an n-dimensional descriptor space into two regions that correspond to two classes, which is widely applied in QSAR modeling [21]. At each stage of the descriptor selection by SW-LDA, F-test was used to control entering or removing descriptors. Here, the default set of F values in the SW-LDA algorithm (F_max_ = 3.84 and F_min_ = 2.71) was adopted.

#### 3.3.2. Support Vector Machines (SVM)

SVM, developed by Vapnik, is a comparatively new and powerful classification technique in statistical learning theories [22]. SVM has gained fast popularity in many fields for its remarkable generalization performance and low risk of overfitting. The basic idea of the SVM classifier is to construct an “optimal separate hyperplane”, which is the one with the maximal margin of separation between the two classes. SVM uses kernel functions to transform input data to become more separable in a high-dimensional feature space. Several kernel functions, such as the radial basis function (RBF), spline and bessel, are available for nonlinear transformation of the input space. The RBF kernel is the most popular kernel functions used in SVM [23]. Here, the Gaussian RBF kernel function was used to perform the non-linear mapping.

The performances of SVM for classification depend on two parameters of RBF kernel function: capacity parameter (C) and γ. C is a regularization penalty parameter, which controls the tradeoff cost between maximizing margins and minimizing training errors. γ controls the generalization ability of SVM. The combination of C and γ should be optimized in order to obtain better results.

#### 3.3.3. Radial Basis Function Neural Network (RBFNN)

Artificial neural networks are general-purpose computing techniques that attempt to solve difficult non-linear problems, like the human brain’s manner of working. Radial basis function neural network (RBFNN) is an artificial neural network that uses RBF as activation functions and its application has increased rapidly in the last few years [24]. RBFNN consists of three layers: an input layer, a hidden layer with a non-linear RBF activation function, and a linear output layer. The input layer only distributes the input vectors to the hidden layer. The hidden layer of RBFNN consists of a number of RBF units (nh) and bias (bk). Each neuron in the hidden layer applies an RBF as a nonlinear transfer function to operate on the input information. The most often-used RBF is the Gaussian function, which is characterized by a center and width. By measuring the Euclidean distance between an input vector (x) and the radial basis function center, the RBF function performs the nonlinear transformation. A detailed description of the theory of RBFNN has been adequately described elsewhere [25].

The performance of a RBFNN is greatly influenced by the number of RBF units (nh, the hidden layer centers). Too low nh gives rise to a poor estimation of relation even in the calibration set, and too many hidden layer neurons causes overfitting [26]. Therefore, the network parameters should be optimized before training.

#### 3.3.4. Classification and Regression Trees (CART)

CART is a non-parametric method introduced by Breiman et al., applicable for exploratory analysis and classification [27]. This classification technique is a form of binary recursive partitioning. The term “binary” means that a set of samples, represented by a “node” in a decision tree, can only be split into two new groups based on splitting criteria i.e., the Gini diversity index, the twoing rule, and the deviance index until, finally, it could no longer model the space.

The CART procedure contains three steps [28]. First an overlarge tree is built. The obtained tree has lots of terminal nodes and describes the training set almost perfectly, but shows a poor predictive ability for new samples. Second, the pruning step corresponds to cutting away branches of the over-large tree to find smaller trees with improved predictive ability, but without losing accuracy. The last step consists of choosing the optimal tree by taking into account its predictive ability. The choice often depends on the results of a cross-validation step.

### 3.4. Model Validation

Internal and external validations were performed to evaluate the predictive ability and reliability of QSAR models. For the internal validation, the leave-one-out (LOO) cross-validation, which produces a number of models by leaving one object from the training set as a validation set once, is often considered as the best way to validate the quality of derived models [29]. Generally, when the value of LOO cross-validated accuracy (accuracy_LOO_) is greater than 50%, the model is acceptable [30]. For the external validation, the prediction accuracy of the external test set was calculated to assess the performance of the obtained model.

All algorithms were accomplished in MATLAB 8.0 and run on a computer (Intel(R) Pentium(R), 2.00 GB RAM).

### 3.5. Performance Measures

Performance of QSAR classification models was often evaluated by the following parameters: overall prediction accuracy (Q), sensitivity (SE), and specificity (SP) [31]. Q is the most common measure of overall performance, which represents the prediction accuracy for active compounds and inactive compounds in the dataset. Q = (T_P_ + T_N_)/(T_P_ + T_N_ + F_P_ + F_N_), where T_P_ is the number of true positives, T_N_ is the number of true negatives, F_P_ is the number of false positives, and F_N_ is the number of false negatives. SE = T_P_/(T_P_ + F_N_) represents the prediction accuracy for the active compounds. while, SP = T_N_/(T_N_ + F_P_) represents the prediction accuracy for the inactive compounds.

### 3.6. Screening New ABCA1 Up-Regulators

One main purpose of our study was to construct highly-predictive QSAR classification models as a fast filter for screening highly-potent ABCA1 up-regulators. Thus, to discover new ABCA1 up-regulators, ZINC database was used [32]. ZINC database contained over 35 million diverse purchasable compounds. Considering that our QSAR models were constructed based on flavanoids, compounds with flavanoid skeletons, especially those with structural similarity to the best active ABCA1 up-regulator (compound **36**) can be well-predicted by our QSAR models. Thereby, the molecular structural similarity between compounds in ZINC database and compound **36** was first calculated using the Tanimoto coefficient in Open Bable 2.3.1 (OpenEye Scientific Software, Santa Fe, NM, USA) [33]. The coefficient is defined as c/(a + b + c), which is the ratio of the atomic pairs that are shared between two compounds divided by their union. The variable c is the number of atomic pairs common in both compounds, while a and b are the numbers of their unique atomic pairs. A good cutoff for the biologically similar molecules is 0.7 or 0.8 [34]. Here, compounds with structural similarity of bigger than 0.7 were selected from the ZINC database. All fit compounds were imported into MOE database for further analysis. Hydrogen atoms and partial charges were assigned, and then they were energy minimized using the molecular mechanics force field method with a convergence criterion of 0.01 kcal/mol. Then, to avoid the limitation or overemphasis of any modeling approach, above obtained QSAR models were combined to generate a consensus model [35], which was applied to screen new highly-potent ABCA1 up-regulator from these compounds.

### 3.7. Exploring the Mechanism of ABCA1 Regulator by Molecular Docking

Further to exploring the mechanism of the ABCA1 up-regulator, molecular docking was employed to study the binding modes and important interactions. It is well-known that transcription of ABCA1 is markedly induced by activation of the nuclear receptor liver X receptor (LXRβ). Potent ABCA1 up-regulators, such as compounds **19** and **36** were found to preferentially activate LXRβ. Therefore, new screened compounds were docked into the ligand-binding site of LXRβ to better understand their up-regulation roles in the expression of ABCA1, and also further screened potent ABCA1 up-regulators by molecular docking embedded in MOE.

The docking simulation was carried out by following steps. First, the three dimension crystal structure of hLXRβ-T0901317 complex (PDB code: 1PQC) was retrieved from the RSCB protein databank (PDB: 1PQC). Then, the protein was protonated using AMBER99 force field and minimized with a RMSD gradient of 0.05 kcal/mol Å. Additionally, the ligand atom mode was utilized to define the binding site, and the docking placement was using triangle matcher algorithm. Finally, two rescoring methods including London dG and Affinity dG, together with a force field were adopted to compute the interactions [36].

## 4. Conclusions

The main goal of this study was to develop QSAR classification models as a potential screening tool for identifying highly-potent ABCA1 up-regulators targeting LXRβ based on a series of new flavonoids by multiple QSAR modeling, structure similarity analysis, and molecular docking. Initially, four different linear and non-linear classification approaches, such as LDA, SVM, CART, and ANN, were applied to construct different QSAR classification models based on SW-LDA-selected optimal descriptors. Satisfactory results were obtained with the introduced methods. The statistical results indicated that the QSAR classification models derived by LDA, SVM, RBFNN, and CART were powerful tools for classifying highly-potent ABCA1 up-regulators, producing the train accuracy of 90.91%, 96.67%, 96.67%, and 100%, respectively, and the test accuracy of 90.91%, 90.91%, 90.91%, and 81.82%, respectively. Additionally, the QSAR study uncovered that a_nCl, lip_don, vsurf_DD23, and vsurf_W2 were important features in defining the up-regulation activity of ABCA1 expression. Then, to avoid the limitation or overemphasis of any modeling approach, a consensus QSAR model was developed by combining the predictions from individual models, which was used to screen highly-potent ABCA1 up-regulators from the ZINC database. Aiming to ensure maximum reliability and accuracy of our QSAR models, molecular similarity analysis was performed to filter compounds in the ZINC database compared to compound **36**. The compounds that fulfilled the requirement of structural similarity of 0.7 were subjected to the consensus QSAR model, which led to the discovery of 50 potential highly-potent ABCA1 up-regulators. Then, to better understand their up-regulation roles in the expression of ABCA1, the molecular docking simulation was applied to docking these 50 compounds into the LXRβ active site. Finally, 10 compounds were found to have larger docking scores for LXRβ than the known highly-potent ABCA1 up-regulator (compound **19**), which was reported to target LXRβ. The molecular binding patterns and docking scores of these 10 molecules also suggested that they could be robust ABCA1 up-regulators targeting LXRβ, which was modulated by hydrogen bonding, hydrophobic, and pi-pi stacking interaction inside the binding pocket. Overall, this information may be used as guidelines for the discovery of novel and robust ABCA1 up-regulators targeting LXRβ.

## Figures and Tables

**Table 1:** Molecular descriptors and the standardized coefficient of the LDA model.

Descriptor	Chemical Meaning	F to Remove	Wilks’ Lambda	Standardized Coefficient
a_nCl	Number of chlorine atoms	7.391	0.479	0.707
lip_don	The number of OH and NH atoms	41.433	0.981	−1.836
vsurf_DD23	Contact distances of lowest hydrophobic energy	4.914	0.442	0.605
vsurf_W2	Hydrophilic volume	14.746	0.587	1.243

**Table 2:** The correlation matrix of descriptors.

Descriptors	a_nCl	lip_don	vsurf_DD23	vsurf_W2
a_nCl	1	0.150	−0.078	−0.047
lip_don	0.150	1	0.330	0.600
vsurf_DD23	−0.078	0.330	1	0.096
vsurf_W2	−0.047	0.600	0.096	1

**Table:**
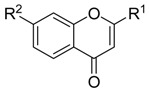


NO.	R^1^	R^2^	Fold Activation	Class ^a^ Exp.	Predicted Class			
					LDA	SVM	RBFNN	CART
1 *	quinolin-2-yl	OH	1.36	0	0	0	0	0
2	naphthalen-2-yl	OH	1.57	0	0	0	0	0
3	1H-indol-3-yl	OH	1.9	1	1	1	1	1
4 *	benzo[b]thiophen-3-yl	OH	1.44	0	0	0	0	0
5	3-phenoxybenzen-1-yl	OH	1.17	0	0	0	0	0
6 *	4-carboxybenzen-1-yl	OH	1.66	0	0	0	0	0
7	(1,1′-biphenyl)-4-yl	OH	1.15	0	0	0	0	0
8	5-(4-methoxyphenyl)thiophen-2-yl)	OH	1.03	0	0	0	0	0
9 *	5-methylfuran-2-yl	NH_2_	1.30	0	0	0	0	0
10	5-methylthiophen-2-yl	NH_2_	1.47	0	0	0	0	0
11 *	4-isopropylbenzen-1-yl	OH	1.22	0	0	0	0	0
12	4-ethoxyphenyl	NH_2_	1.37	0	0	0	0	0

**Table:**
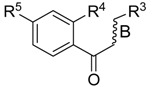


NO.	R^3^	R^4^	R^5^	B	Fold Activation	Class ^a^ Exp.	Predicted Class			
							LDA	SVM	RBFNN	CART
13	5-methylfuran-2-yl	OH	NH_2_	d	2.09	1	1	1	1	1
14	5-methylthiophen-2-yl	OH	NH_2_	d	2.00	1	1	1	1	1
15	4-ethoxyphenyl	OH	NH_2_	d	1.92	1	1	1	1	1
16	4-(methylthio)phenyl	OH	NH_2_	d	1.26	0	0	0	0	0
17 *	3-methoxyphenyl	OH	NH_2_	d	2.09	1	1	1	1	1
18	4-fluorophenyl	OH	NH_2_	d	1.36	0	0	0	0	0
19	4-chlorophenyl	OH	NH_2_	d	1.16	0	0	0	0	0
20	3,5-bis(trifluoromethyl)-phenyl	OH	NH_2_	d	1.12	0	0	0	0	0
21*	5-methylfuran-2-yl	OH	NH_2_	s	1.90	1	1	1	1	1
22	3,4,5-trimethoxyphenyl	OH	NH_2_	d	0.98	0	1	0	0	0
23	3,4-dimethoxyphenyl	OH	NH_2_	d	1.86	1	0	0	0	1
24	4-isopropylphenyl	OH	NH_2_	d	2.05	1	1	1	1	1
25	4-ethylphenyl	OH	NH_2_	d	1.73	1	1	1	1	1
26	(1,1'-biphenyl)-4-yl	OH	NH_2_	d	1.55	0	0	0	0	0
27 *	3-phenoxybenzen-1-yl	OH	NH_2_	d	1.76	1	1	1	1	1
28	5-(4-methoxyphenyl)-thiophen-2-yl)	OH	NH_2_	d	1.09	0	0	0	0	0
29	benzo[*b*]thiophen-3-yl	OH	NH_2_	d	2.31	1	1	1	1	1
30 *	1*H*-indol-3-yl	OH	NH_2_	d	1.76	1	1	1	1	0
31	naphthalen-2-yl	OH	NH_2_	d	2.64	1	1	1	1	1
32	benzo[*d*][1,3]dioxol-5-yl	OH	NH_2_	d	1.92	1	1	1	1	1
33	5-methylfuran-2-yl	OCH_3_	N(CH_3_)_2_	d	1.57	0	0	0	0	0
34	naphthalen-2-yl	OCH_3_	N(CH_3_)_2_	d	1.35	0	0	0	0	0
35 *	naphthalen-2-yl	OH	NH_2_	s	1.34	0	1	1	1	1
36	naphthalen-2-yl	OCH_3_	NH_2_	d	0.88	0	0	0	0	0
37	5-methylfuran-2-yl	OCH_3_	NH_2_	d	1.20	0	0	0	0	0
38	5-methylthiophen-2-yl	OCH_3_	NH_2_	d	1.50	0	0	0	0	0
39	4-ethoxyphenyl	OCH_3_	NH_2_	d	1.06	0	0	0	0	0
40	5-methylfuran-2-yl	OH	N(CH_3_)_2_	d	1.02	0	1	0	0	0
41 *	5-methylthiophen-2-yl	OH	CH_3_CONH	d	1.08	0	0	0	0	0

* Test set; ^a^ 1 denotes high active compounds, 0 denotes low active compounds; in column “B”, s denotes single bond, d denotes double bond.

**Table 4:** The classification performance of four different modeling approaches.

Model	Accuracy_train_	Accuracy_test_	Accuracy_LOO_	Total Accuracy	Sensitivity	Specificity
LDA	90%	90.91%	83.33%	90.24%	90%	90%
SVM	96.67%	90.91%	86.67%	95.12%	90%	100%
ANN	96.67%	90.91%	83.33%	95.12%	90%	100%
CART	100%	81.82%	83.33%	95.12%	100%	100%
Consensus	96.67%	90.91%	83.33%	95.12%	90%	100%

Accuracy_train_ represents predictive classification accuracy of the training set; Accuracy_test_ represents predictive classification accuracy of the test set; Accuracy_LOO_ represents predictive classification accuracy of LOO cross-validation of the model. Total accuracy represents total classification accuracy of training and test sets.

**Table 5:** Chemical structures of newly-screened ABCA1 up-regulator targeting LXRβ.

NO.	Structure	Docking Score	Similarity
Compound **36**	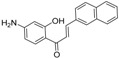	−10.997	1
Compound **19**	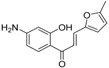	−8.451	0.7
ZINC39949652	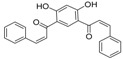	−13.255	0.725
ZINC08665430	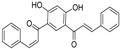	−14.001	0.725
ZINC3250227	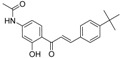	−11.625	0.756
ZINC32502236	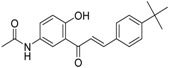	−11.277	0.7
ZINC05777271	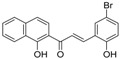	−11.0604	0.723
ZINC32502232	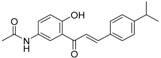	−10.194	0.7
ZINC05173700	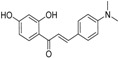	−10.0822	0.8
ZINC05211016	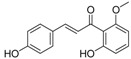	−9.8112	0.707
ZINC32502231	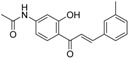	−9.3668	0.756
ZINC32502229	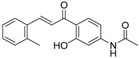	−9.1047	0.747
